# Abacavir safety and effectiveness in young infants with HIV in South African observational cohorts

**DOI:** 10.1177/13596535231168480

**Published:** 2023-02

**Authors:** Reneé de Waal, Helena Rabie, Karl-Günter Technau, Brian Eley, Nosisa Sipambo, Mark Cotton, Andrew Boulle, Robin Wood, Frank Tanser, Geoffrey Fatti, Matthias Egger, Mary-Ann Davies, International epidemiology Databases to Evaluate AIDS (IeDEA) Southern Africa

**Affiliations:** 1 Centre for Infectious Disease Epidemiology and Research, University of Cape Town, Cape Town, South Africa; 2 Department of Paediatrics and Child Health, University of Stellenbosch and Tygerberg Hospital, Tygerberg, South Africa; 3 Empilweni Services and Research Unit, Rahima Moosa Mother and Child Hospital, Department of Paediatrics and Child Health, University of the Witwatersrand, Johannesburg, South Africa; 4 Paediatric Infectious Diseases Unit, Red Cross War Memorial Children’s Hospital, Cape Town, South Africa; 5 Department of Paediatrics and Child Health, University of Cape Town, Cape Town, South Africa; 6 Department of Paediatrics and Child Health, University of the Witwatersrand, Chris Hani Baragwanath Academic Hospital, Johannesburg, South Africa; 7 The Desmond Tutu HIV Centre, University of Cape Town, Cape Town, South Africa; 8 Africa Health Research Institute, Mtubatuba, South Africa; 9 Kheth’Impilo AIDS Free Living, Cape Town, South Africa; 10 Division of Epidemiology and Biostatistics, Department of Global Health, Stellenbosch University, Stellenbosch, South Africa; 11 Institute of Social and Preventive Medicine, University of Bern, Bern, Switzerland

**Keywords:** abacavir, HIV, infants, effectiveness, observational cohort

## Abstract

**Background:**

WHO guidelines recommend abacavir in first-line antiretroviral treatment for children and neonates. However, there is no approved dose <3 months of age, and data in neonates are limited.

**Methods:**

We included infants who initiated ART aged <3 months, between 2006 and 2019, in nine South African cohorts. In those who received abacavir or zidovudine, we described antiretroviral discontinuation rates; and 6- and 12-month viral suppression (<400 copies/mL). We compared infants aged <28 and ≥28 days, those weighing <3 and ≥3 kg.

**Results:**

Overall 837/1643 infants (51%) received abacavir and 443 (27%) received zidovudine. Median (interquartile range, IQR) age was 52 days (23–71), CD4 percentage was 27.9 (19.2–38.0), and weight was 4.0 kg (3.0–4.7) at ART initiation. In those with ≥1 month’s follow-up, 100/718 (14%) infants discontinued abacavir, at a median of 17.5 months (IQR 6.5–39.5). Abacavir discontinuations did not differ by age or weight category *(p* = 0.4 and 0.2, respectively); and were less frequent than zidovudine discontinuations (adjusted hazard ratio 0.14, 95% confidence interval 0.10–0.20). Viral suppression at 12 months occurred in 43/79 (54%) and 130/250 (52%) of those who started abacavir aged <28 and ≥28 days, respectively *(p* = 0.8); 11/19 (58%) and 31/60 (52%) in those who weighed <3 and ≥3 kg, respectively *(p* = 0.6); and 174/329 (53%) in those on abacavir versus 77/138 (56%) in those on zidovudine (adjusted odds ratio 1.8, 95% confidence interval 1.0–3.2).

**Conclusion:**

Our data suggest that abacavir may be used safely in infants <28 days old or who weigh <3 kg.

## Background

Local and international guidelines for early infant HIV diagnosis have progressively included birth PCR testing for all HIV-exposed infants, and antiretroviral treatment (ART) guidelines have advanced to immediate ART initiation for all,^1,2^ which has improved outcomes for infants with HIV.^3–5^ However, mortality and loss to follow-up remains relatively high,^3,6,7^ and viral suppression rates are lower than in older children.^3,8,9^ Limited ART options in this vulnerable population might play a role in these poorer outcomes.^3,10^

Currently zidovudine, abacavir, and lamivudine are the only nucleoside reverse transcriptase inhibitors (NRTIs) recommended in World Health Organization (WHO) guidelines for neonates.^1^ Overall, abacavir is relatively well tolerated and efficacious compared with alternative nucleoside reverse transcriptase inhibitors.^11^ The incidence of abacavir hypersensitivity reaction is lower in Sub-Saharan Africa than elsewhere.^12^ WHO guidelines recommend abacavir as part of the preferred first-line ART regimen in children and neonates^1^. However dosing under 3 months of age has not been approved by local and international regulatory agencies,^13^ and data regarding safety and effectiveness in neonates are limited.

We describe the safety and effectiveness of abacavir in infants aged younger than 3 months using routine clinical data from nine South African cohorts participating in the International epidemiology Databases to Evaluate AIDS collaboration.

## Methods

### Study population and setting

The study sites represent primary and hospital level public sector clinics in three provinces, and a national private sector cohort. All sites collect routine clinical data electronically, and treat patients according to South African National Department of Health guidelines.^2^ Since 2010, infants less than 12 months old have been eligible for ART regardless of CD4 count.^14^ Abacavir was part of the preferred first-line regimen for all infants and children younger than 3 years from 2010 until 2019, although no dosing recommendations were provided for neonates (infants younger than 28 days) or infants weighing less than 3 kg, and consultation with a neonatal antiretroviral prescribing expert was recommended.^2,14,15^ Before 2010, infants were eligible for ART if their CD4 percentage was less than 20, and stavudine-based regimens were recommended.^16^ We included infants who initiated ART before 3 months of age between 2006 and 2019, irrespective of starting regimen.

### Data management and analysis

Sites exported data using a standard data transfer format and checked their records for missing reasons for stopping or switching any antiretroviral drugs where possible. We described abacavir use relative to other ART regimens recommended over the time period of the study. We described the proportion of infants with at least 1 month’s follow-up who discontinued abacavir for at least 30 days, and used Cox regression to compare abacavir and zidovudine discontinuations. We estimated the proportion of infants with viral load less than 50 and less than 400 copies/mL at six and 12 months after abacavir initiation. We compared infants initiating abacavir aged less than 28 days with those aged 28 days and older, and those who weighed less than 3 kg with those who weighed 3 kg and more. We also compared viral suppression in infants who started abacavir with those who started zidovudine, using logistic regression. We excluded stavudine from the comparisons of discontinuations and viral suppression as it is no longer recommended in guidelines.

## Results

We included 1,643 infants who started ART before 3 months of age: 775 (47%) started abacavir in their first ART regimen, 62 (4%) switched to abacavir from another NRTI before 3 months of age; 443 (27%) started zidovudine and did not switch to abacavir before 3 months; and 363 (22%) started stavudine. Their characteristics are described in Table 1. Median duration of follow-up was 19.8 months (interquartile range, IQR 5.3–46.6) in those on abacavir, and 11.2 months (IQR 1.7–35.4) in those on zidovudine. Infants receiving abacavir were older at the time of ART initiation, and were more likely to be on a protease inhibitor-based regimen, than those on zidovudine. By the end of follow-up, 37 (4%) and 39 (9%) infants had died, 407 (49%) and 221 (50%) had transferred to other facilities, and 106 (13%) and 29 (7%) were lost to follow up, for abacavir and zidovudine, respectively.

### Antiretroviral discontinuations

**Figure 1.**
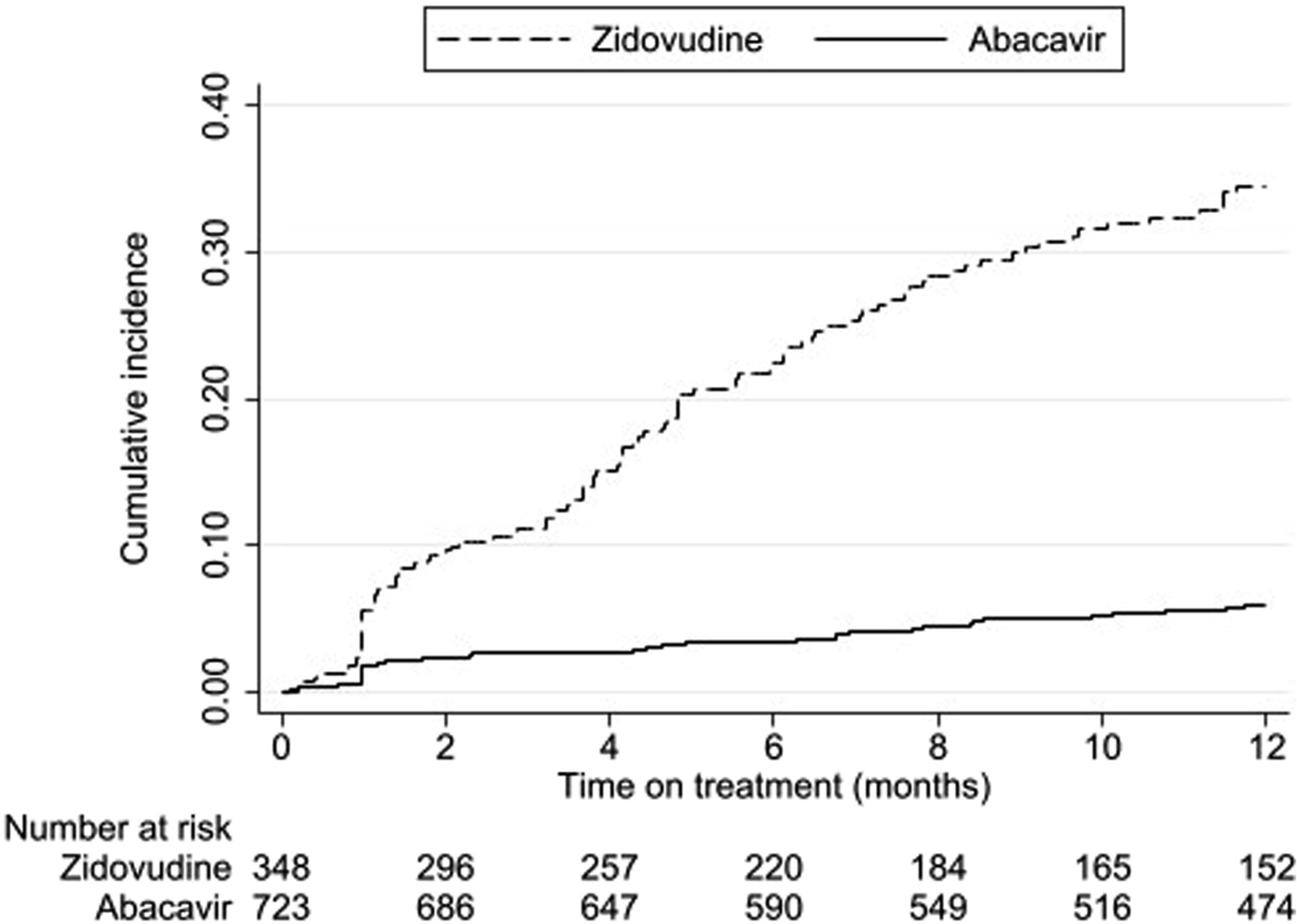
Abacavir or zidovudine discontinuations during the first year of treatment.

In those with at least 1 month’s follow-up, 100/718 (14%) infants discontinued abacavir for at least 30 days, at a median of 17.5 months (IQR 6.5–39.5) after abacavir initiation. Forty-six of those infants later restarted abacavir. Reasons for abacavir discontinuations were documented in 33 infants: treatment failure in 8, drug stock-outs in 8, hypersensitivity in 1, lipodystrophy in 1, drug interaction in 1, and physicians’ decision (not otherwise specified) in 14. The infant with hypersensitivity was male and started abacavir at 73 days old, weighing 4.3 kg. The abacavir discontinuation was documented at 130 days (but may have occurred earlier). There were no significant differences in the proportion of discontinuations by age or weight category *(p* = 0.4 and 0.2, respectively, Additional File 1). Results were similar when analysis was restricted to the 54 infants not restarting abacavir by the end of their follow-up period. Discontinuations were less frequent with abacavir than zidovudine (hazard ratio 0.14, 95% confidence interval 0.10 to 0.20, adjusted for protease inhibitor versus non-nucleoside reverse transcriptase inhibitor, age at ART initiation, and year of ART initiation, Figure 1).

### Viral suppression

**Figure 2.**
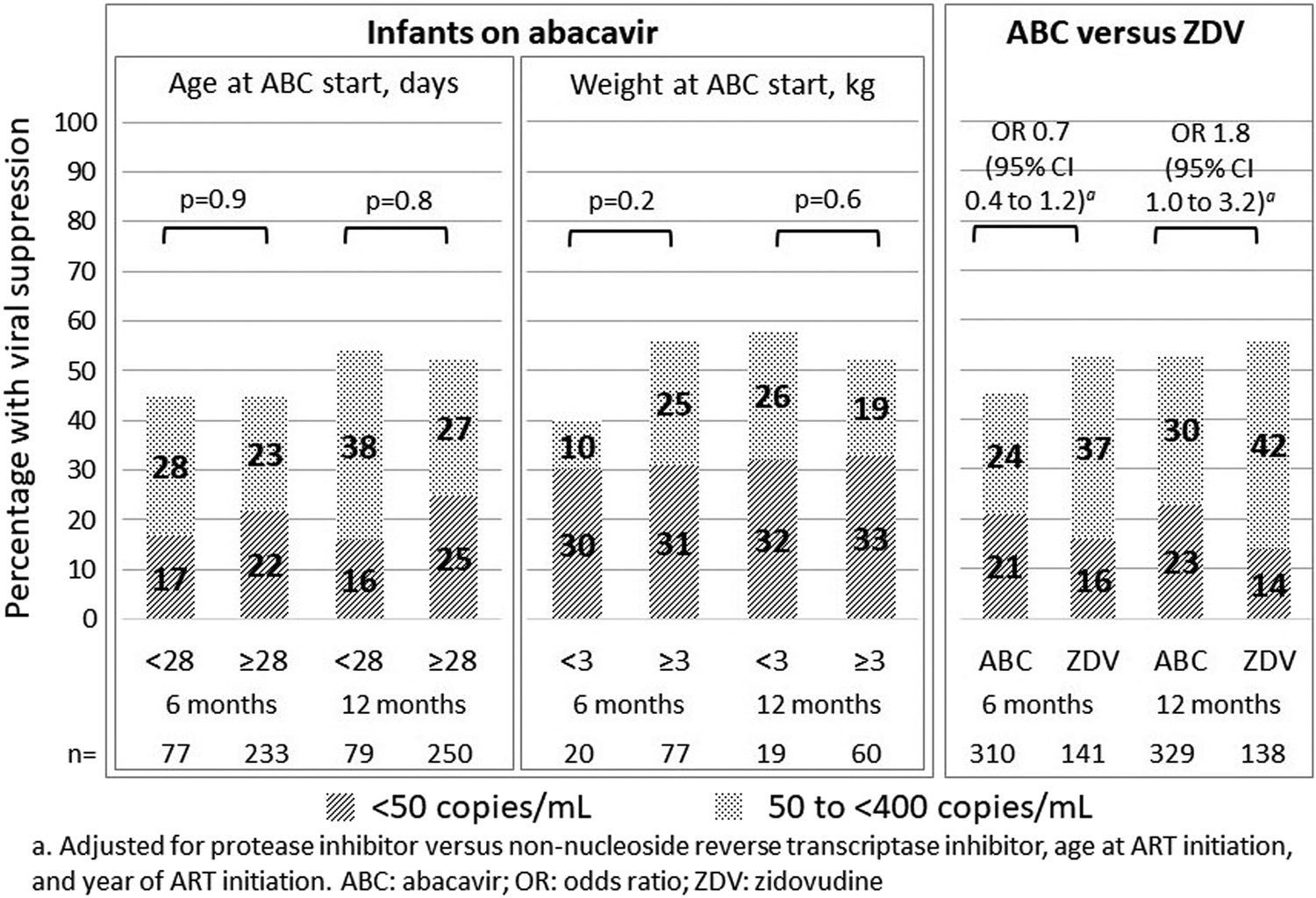
Viral suppression at 6 and 12 months by age and weight categories in infants on abacavir, and abacavir compared with zidovudine.

Viral load was measured at 6 months (within 4–8 months) in 310/615 (50%) infants on abacavir (regardless of whether or not they started on a different NRTI and switched to abacavir by 3 months of age), and 141/280 (50%) infants on who started a zidovudine based regimen and remained on zidovudine, with at least 6 months’ follow-up. Viral load was measured at 12 months (within 8–18 months) in 329/505 (65%) infants on abacavir, and 138/215 (64%) infants on zidovudine, with at least 12 months’ follow-up. There were no significant differences in the proportion with viral load less than 400 copies/mL by age or weight category, or for abacavir versus zidovudine (Figure 2). Results were similar when we restricted the analysis to infants on abacavir who hadn’t previously received another NRTI (Additional File 2).

## Discussion

In our cohort of 837 infants who started abacavir aged less than 3 months, abacavir discontinuations, and viral suppression at 6 and 12 months, were not significantly different in infants aged less than 28 days than those 28 days and older, or in infants who weighed less than 3 kg, than those who weighed 3 kg or more. Viral suppression was similar in infants who received abacavir or zidovudine.

Viral suppression rates were relatively low compared with some previous studies, possibly because of differences in patient populations, or challenges with ART medicine formulations or side effects. A cohort of infants on abacavir from Europe reported that 64/92 (70%) and 59/77 (77%) had viral loads <400 copies/mL at 6 and 12 months, respectively.^17^ However, the rates seen in our study are similar to those from a previous Southern Africa study, where most infants were on stavudine-based ART. This study reported a 56% probability of viral load <400 copies/mL at 12 months,^5^ while a Kenyan study reported a viral load <250 copies/mL at 6 months in 32% of infants.^9^

We were unable to report all adverse drug reactions as they were not recorded routinely, but tried to identify those that resulted in treatment discontinuation. Reasons for discontinuations were only available for a third of children. Nonetheless, that only one incident of hypersensitivity was reported is reassuring. Discontinuations were less frequent with abacavir than zidovudine, probably mainly because of clinicians switching infants from zidovudine to abacavir as they got older, in keeping with guidelines that recommended abacavir use for infants aged >4 weeks. We were also unable to assess other factors that might influence viral suppression, such as adherence.

Our routine data did not include abacavir doses, although clinicians at the sites followed WHO dosing guidelines, which recommended 8 mg/kg twice daily in infants aged 28 days and older.^18^ Folder review at one participating site confirmed that most infants received this dose.^19^ Pharmacokinetic studies in 25 infants living with HIV at a median age of 6 weeks, and in 10 HIV-exposed uninfected infants aged 15 days or less showed that doses of 8 mg/kg gave exposures higher than those in older infants but were safe and well tolerated.^20,21^ Current WHO guidelines include dolutegravir as part of the preferred regimen for infants, but South African guidelines at the time of the study did not include dolutegravir for this population, so we were unable to assess outcomes of infants on abacavir and dolutegravir.^2^

Despite the limitations described, data from our relatively large cohort of infants provides reassuring evidence regarding the use of abacavir in infants aged less than 3 months.

## Conclusion

Data regarding abacavir use in neonates are limited. Our study suggests that abacavir may be used effectively, and did not result in substantial treatment discontinuations, in infants younger than 3 months, including in those less than 28 days old, or who weigh less than 3 kg.

## Supplementary Material

Supplementary material_ABC in infants

## Figures and Tables

**Table 1.** Characteristics at abacavir, zidovudine, or stavudine initiation.

	Abacavir	Zidovudine	Stavudine
*n*	837	443	363
Male, *n* (%)	344 (41%)	218 (49%)	183 (50%)
Median (IQR) age, days	52 (23–71)	17 (4–57)	68 (52–81)
Age <28 days, *n* (%)	232 (28%)	270 (61%)	26 (7%)
Median CD4% (IQR), (*n* = 328, 238)	27.9 (19.2–38.0)	37.0 (22.9–51.0)	22.2 (12.8–32.6)
Median log_10_ viral load (IQR), (*n* = 298, 259,155)	5.9 (4.8–6.6)	5.0 (4.0–6.0)	6.0 (5.3–6.5)
Median (IQR) weight, kg (*n* = 247,109,171)	4.0 (3.0–4.7)	3.3 (2.9–4.0)	3.7 (3.1–4.3)
Weight<3 kg, *n* (%)	53 (6%)	35 (8%)	32 (9%)
Protease inhibitor-based regimen,^1^*n* (%)	756 (90%)	209 (47%)	343 (94%)
ART period, *n* (%)			
<2010	23 (3%)	123 (28%)	310 (85%)
2010–2015	616 (74%)	158 (36%)	49 (13%)
>2015	198 (24%)	162 (36%)	4 (1%)

ART, antiretroviral treatment; IQR, interquartile range.

1 Protease inhibitor was ritonavir-booster lopinavir in 99% of infants; non-nucleoside reverse transcriptase inhibitor was nevirapine in 92% of infants.
