# Supplementary material for: Abacavir safety and effectiveness in young infants with HIV in South African observational cohorts
Source: Antivir Ther. Author manuscript; Available in PMC 2024 Mar 25. (PMC10961679; doi:10.1177/13596535231168480)
Supplement: Supplementary material_ABC in infants [file NIHMS1978024-supplement-Supplementary_material_ABC_in_infants.pdf]

Additional file 1. Abacavir discontinuations by age and weight categories in infants on abacavir with at least one month's follow up

	Weight at abacavir initiation (kg)						
	**<3.0**	**3.0–3.9**	**4.0–4.9**	**5.0–5.9**	**>6.0**	**Missing**	**Total**
**Overall**							
n	39	46	55	27	14	537	718
Discontinuations (%)	4 (10)	5 (10)	10 (18)	0	3 (21)	78 (15)	100 (14)
**Age at abacavir start <28 days**							
n	16	12	7	3	1	164	203
Discontinuations (%)	1 (6)	1 (8)	2 (29)	0	0	19 (12)	24 (12)
**Age at abacavir start 28 days to 3 months**							
n	23	34	48	24	13	373	515
Discontinuations (%)	3 (13)	4 (12)	8 (17)	0	2 (15)	59 (16)	76 (15)

Additional file 2. Viral suppression at 6 and 12 months by age and weight categories in infants on abacavir (excluding those who switched from another NRTI), and abacavir compared with zidovudine.

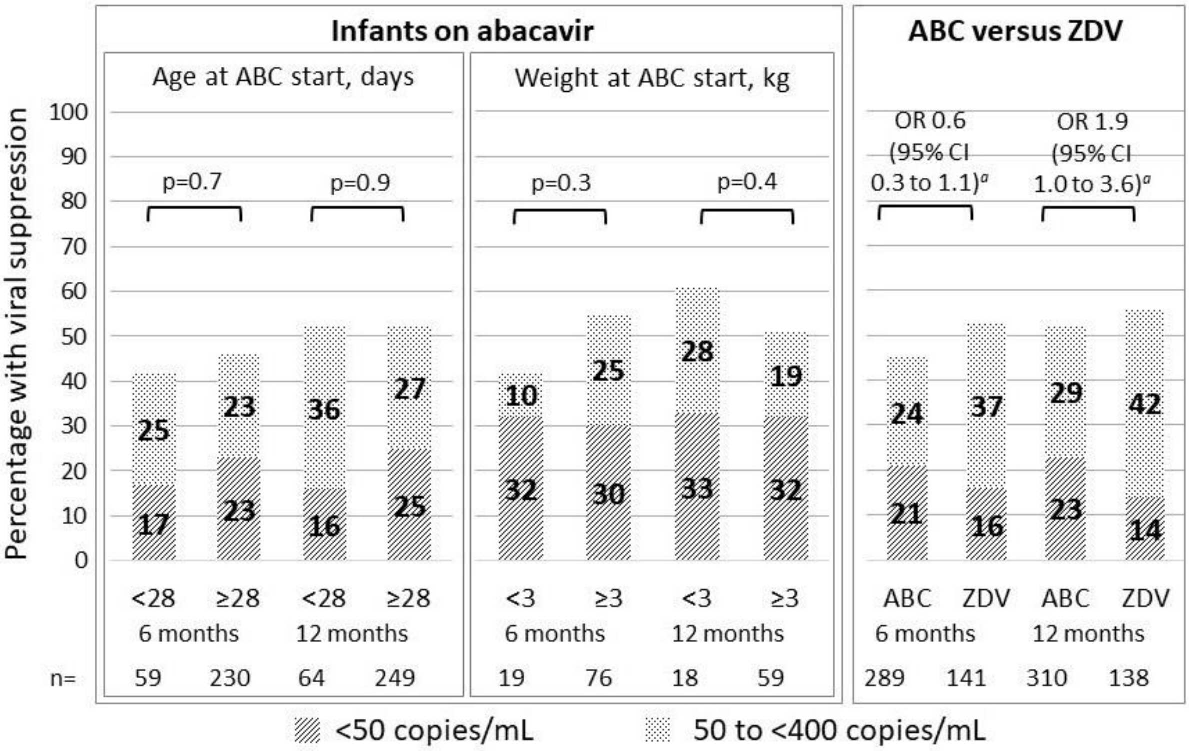

a. Adjusted for protease inhibitor versus non-nucleoside reverse transcriptase inhibitor, age at ART initiation, and year of ART initiation. ABC: abacavir; OR: odds ratio; ZDV: zidovudine

Viral load was measured at 6 months (within 4–8 months) in 289/570 (51%) infants on abacavir, and 141/280 (50%) infants on zidovudine, with at least 6 months' follow up. Viral load was measured at 12 months (within 8–18 months) in 310/473 (66%) infants on abacavir, and 138/215 (64%) infants on zidovudine, with at least 12 months' follow up. There were no significant differences in the proportion with viral load less than 400 copies/mL by age or weight category, or for abacavir versus zidovudine.
